# Using death certificate data to study place of death in 9 European countries: opportunities and weaknesses

**DOI:** 10.1186/1471-2458-7-283

**Published:** 2007-10-08

**Authors:** Joachim Cohen, Johan Bilsen, Guido Miccinesi, Rurik Löfmark, Julia Addington-Hall, Stein Kaasa, Michael Norup, Gerrit van der Wal, Luc Deliens

**Affiliations:** 1 End-of-Life Care Research Group, Vrije Universiteit Brussel, Brussels, Belgium; 2 Centre for Environmental Philosophy and Bioethics, Ghent University, Ghent, Belgium; 3 Centre for Study and Prevention of Cancer, Florence, Italy; 4 Centre for Bioethics, LIME, Karolinska Institutet and Uppsala University, Stockholm Sweden; 5 School of Nursing and Midwifery, Southampton University, UK; 6 Norwegian University of Science and Technology Trondheim, Norway; 7 Department of Medical Philosophy and Clinical Theory, University of Copenhagen, Denmark; 8 VU University Medical Center, Department of Public and Occupational Health, EMGO Institute, Amsterdam, the Netherlands

## Abstract

**Background:**

Systematic and reliable epidemiological information at population level, preferably cross-national, is needed for an adequate planning of (end-of-life) health care policies, e.g. concerning place of death, but is currently lacking. This study illustrates opportunities and weaknesses of death certificate data to provide such information on place of death and associated factors in nine European countries (seven entire countries and five regions).

**Methods:**

We investigated the possibility and modality of all partners in this international comparative study (BE, DK, IT, NL, NO, SE, UK) to negotiate a dataset containing all deaths of one year with their national/regional administration of mortality statistics, and analysed the availability of information about place of death as well as a number of clinical, socio-demographic, residential and healthcare system factors.

**Results:**

All countries negotiated a dataset, but rules, procedures, and cost price to get the data varied strongly between countries. In total, about 1.1 million deaths were included. For four of the nine countries not all desired categories for place of death were available. Most desired clinical and socio-demographic information was available, be it sometimes via linkages with other population databases. Healthcare system factors could be made available by linking existing healthcare statistics to the residence of the deceased.

**Conclusion:**

Death certificate data provide information on place of death and on possibly associated factors and confounders in all studied countries. Hence, death certificate data provide a unique opportunity for cross-national studying and monitoring of place of death. However, modifications of certain aspects of death certificate registration and rules of data-protection are perhaps required to make international monitoring of place of death more feasible and accurate.

## Background

There are several reasons why it is important for public health policy to study place of death and to gain a better understanding of the reasons why people die where they die. The place of death is often regarded as an important parameter for the quality of the end-of-life [1,2], and there seems to be a large discrepancy between the preferred and actual place of death [3,4]. Moreover, as allocation of means is becoming increasingly important in healthcare organisation, and as healthcare costs are particularly high at the end-of-life [5-7], there can be economical motives. The UK for instance has made policy incentives to allow more people to die at home if they want to, explicitly referring to cost-saving effects of home deaths [8]. Many other countries implemented policy measures to reduce the number of acute care hospitalisations as a means to restrict hospital expenditure [9,10].

However, there are a number of deficiencies of place of death research which make it difficult to compare results and to draw meaningful conclusions. Previous research has often been limited with regard to sample size [11-13], patient population [11,12,14-27] (e.g. only cancer patients, or only patients in a palliative care program) or setting [12,13,16,18,19,22,23,25,27,28] (e.g. only in a home situation), and often did not use appropriate multivariable statistical models allowing sufficient adjustment for confounders. Because reliable epidemiological data are necessary for planning, organisation and implementation of (end-of-life) health care policies, the challenge is to develop systematic and comprehensive information at population level [29], eventually serving for cross-national comparisons,.

Although employed for several studies in USA [19,30-36], UK [37,38], Japan [39], Italy [40], Denmark [41], and Belgium [42], death certificate data remain underexplored and underexploited in this context. We wanted to examine these opportunities on the basis of national/regional death certificate data in nine European countries (Belgium, Denmark, Italy, The Netherlands, Norway, Sweden, England, Scotland, Wales), collected within the framework of a collaborative end-of-life care research project ("Dying Well in Europe") among seven European partners (BE, DK, IT, NL, NO, SE, UK). The research questions we tried to answer in this article were:

First, what procedure is required to obtain a database of all deaths of one year containing place of death information as well as a number of possibly associated factors, and are there rules that limit the use of the data?

Second, how well do the death certificate data allow describing place of death, and possibly associated factors, indicated as relevant in the literature?

Finally, we will make some recommendations.

## Methods

### Design

In the course of 2005 and 2006 a database was collected containing all deaths of the most recent year for seven entire countries (Denmark, The Netherlands, Norway, Sweden, England, Wales, Scotland), two regions in Belgium (Flanders and Brussels, the Walloon region being left out due to a serious lacking behind in death certificate registration), and three regions in Italy (Emilia Romagna, Tuscany, Milan). All these regions have an autonomous public health policy and authority over the death certificate data.

Besides the place of death, we aimed to include a limited number of clinical, socio-demographic, residential and health care system factors, based on factors identified as relevant in the literature. We therefore drafted a typical database (table 1), based on recommendations from all participants to the study. All partners of the study negotiated a dataset maximally resembling this typical database with their national or regional administration of mortality statistics, which was to be integrated in one common European database on deaths. In case variables of the typical database were not available directly via the mortality statistics, partners needed to inquire for possibilities to combine the data register with other registers.

**Table 1:** Typical aimed data base, to be negotiated with national or regional administration of mortality statistics

1. Year of registration	2003
	
2. Population	all deaths (except stillborns) in the whole country/region
	
3. Variables	Place of death (hospital, care home, home, other, unknown)
	Natural vs. non-natural death
	Underlying cause of death (ICD-coded)
	Age
	Sex
	Civil status
	Living situation/family type
	Level of education
	Place (country) of birth/Nationality
	Municipality of death
	Municipality of residence (ZIP code)
	(area statistics to be linked to place of residence)

### Analysis

Characteristics of the data collection and the collected data will be described:

- procedure to obtain the data

- most recent year available and total number of deaths in the data file

- place of death information

- other variables, potentially associated with place of death, available on the death certificate data, or included via linkage of the death certificate data with other data files.

## Results

### Procedure to obtain the data

There were considerable variations in the procedure to obtain the requested data in the different countries (table 2). Approval of the project, based on a provided project description, by the agencies responsible for the death certificate data was sufficient to get the data files in Italy, Belgium, and Scotland. In the Netherlands this was also the case, but the office's data protection policy to prevent possible identification of individuals implied some restrictions in the use of the data, so that some variables could not be provided (e.g. marital status, place of residence), while aggregations needed to be made for others (e.g. age, cause of death). Data were provided relatively fast in these countries.

**Table 2:** Procedure to obtain the databases

	approval data agency	approval data protections agency	other approval/license	restrictions in use
Belgium*	X			
Denmark	X	X	X	X^‡^
Italy*	X			
Sweden	X		X	X^†^
The Netherlands	X			X
Scotland (UK)	X			
England/Wales (UK)	X	X		
Norway	X	X	X	X

*: comprises all separate regions for Italy, and Belgium

†: data cannot leave European Union

‡: Danish data could initially only be used on-site. After an additional approval of access to micro data, the data could be accessed via internet on one personal computer in Denmark, with the data staying on the server of Statistics Denmark.

In other countries additional approvals were required next to those by the agencies responsible for the death certificate data: in Sweden by the National Board of Health and Welfare; in Denmark by the Danish Data Protection Agency (including an additional approval of access to micro data with a restriction to use the data only within Denmark); in England/Wales by the Micro release panel; and in Norway by the data protection agency and by the Social- and Public Health Department of the Ministry of Health.

In Denmark, Sweden, England/Wales and Norway, the time from the order of the data to the delivery exceeded (sometimes considerably) 6 months. No charges were asked for the datasets in Belgium (Flanders and Brussels), and Italy (all three regions). In the Netherlands, England/Wales and in Scotland the cost price was less than 1,000 euros, in Sweden over 2,500 euros and in Denmark over 3,500 euros.

### Most recent year available and total number of deaths on the data file

The most recent year of the available full and error-checked databases -at the time of the initiation of the study (September 2004)- was 2003 for Flanders(BE), Brussels(BE), The Netherlands, Norway, Scotland (UK), and England/Wales (UK); 2002 for Tuscany (IT), Emilia Romagna (IT), the city of Milan (IT), and Sweden, and 2001 for Denmark.

Total number of deaths ranged from 10,108 in Brussels to 505,341 in England (table 3).

**Table 3:** Total number of deaths on the datafile and year of registration

	year	total number of deaths*
Brussels (Belgium)	2003	10 108
Flanders (Belgium)	2003	57 156
The Netherlands	2003	141 936
Scotland (UK)	2003	58 473
England (UK)	2003	505 341
Wales (UK)	2003	33 810
Norway	2003	42 550
Tuscany (Italy)	2002	39 955
Emilia Romagna (Italy)	2002	45 647
Milan (Italy)	2002	14 247
Sweden	2002	95 064
Denmark	2001	58 355

*: these are all deaths on the datafile, excluding stillbirths

### Place of death information

The categories of the place of death variable on the death certificate data file corresponded in most countries with the categories that could be marked on the actual death certificates (table 4). In the Netherlands it was most comprehensive, comprising the categories: hospital, psychiatric hospital, nursing home, home for older people, other institute, own home, and other. On the Belgian file, the place of death was divided in hospital, care home (which covers both nursing homes and homes for older people), home and other (subdivided in workplace, public road, or a textual specification by the physician). The Scotland data file comprised hospital (hospital, and joint user), care homes (residential homes, nursing homes, and contracturals), own home, other institution (prison, and homes), and other. The England and the Wales file distinguished hospitals, psychiatric hospitals, care homes (residential homes, and nursing homes), own home, and NHS and private hospices. In Norway hospitals and care homes were grouped in a same category on the death certificate.

**Table 4:** Available categories of the variable 'place of death' on the death statistics database

	Hospital	Psychiatric hospital	Nursing home	Home for older people	Other institutes	Home	Other
Belgium^‡^	X		X*			X	X
Denmark	X		X^†^			/	X
Italy^‡^	/					X	X
Sweden	X^¶^	X				/	X
The Netherlands	X	X	X	X	X	X	X
England & Wales (UK)	X	X	X	X	X	X	X
Scotland (UK)	X	X	X	X	X	X	X
Norway	X**	X	X**			X	X

An X indicates categories on the death certificate and on the data file; an/indicates categories on the death certificate but not coded on the datafile

*: One category of 'care home' is given, comprising both nursing homes and homes for older people

†: This is a category 'institution' (comprising all institution, except hospitals)

‡: Comprises all separate regions for Italy and Belgium

¶ not recorded on the death certificate data file, but could be deduced from the postcodes

**: care homes and hospitals are lumped together in one category

However, in three countries the place of death variable on the dataset did not contain all categories that could be marked by certifying physicians on the death certificate [see Additional file 1]. The Italian datasets only made a distinction between 'home' and 'other', while the category 'hospital' from the death certificate was not recorded. The Danish data file only distinguished 'hospitals', 'institutions (but not hospitals)', and 'other', while 'home' could also be marked on the death certificate. In Sweden, place of death, while a certified variable, was not even recorded at all on the death certificate data file. However, 'hospital', 'psychiatric hospital' or 'other' could be deduced from the postcodes of the parish of death, as these institutions have their own postcodes.

### Other variables, potentially associated with place of death

In Belgium and in Italy most desired clinical and socio-demographic information was directly available via the countries' death certificate data (table 5). In other countries the clinical and socio-demographic information directly available via the death certificate data was more limited, but in several countries linkages could be made (via unique identifiers) with other population databases. The living environment of the deceased was however not available in Norway, Sweden, and Scotland. In England and Wales the living environment and the civil status of the deceased, recorded in census data, were not linked to the death certificate data due to privacy rules. The level of education could not be retrieved in The Netherlands and in the UK, but in England, Wales and Scotland the social class based on the last occupation (i.e. NS-SEC code) was available for all deaths below 75 years.

**Table 5:** variables potentially associated with place of death on the data-file

	BE*	DK	IT*	SE	NL	Engl./Wales (UK)*	Scotland (UK)	NO
**Sociodemographic variables**								
age	X	X	X	X	X	X	X	X
sex	X	X	X	X	X	**X**	**X**	X
civil status	X	L	X	X	^†^		**X**	X
living environment/family type of deceased (alone, institute,...)	X	L			L			
level of education	X	L	X	L				L
nationality	X	L	X	X	L	**X**^‡^		L
**Clinical characteristics**								
natural vs. non-natural death	X	X	X	X	X	**X**	**X**	X
cause of death	X	X	X	X	X	**X**	**X**	X
**Residence characteristics**								
municipality of death	X	X	X	X	^†^		**X**	X
municipality of residence	X	X	X	X	^†^	**X**	**X**	X
urbanisation	L	X	L	L	L	**L**	**L**	X
contextual SES	L	L	L	L	L	**L**	**L**	L
hospital bed rate	L	L	L	L	L	**L**	**L**	L

An X indicates that the variable is on the death certificate and on the death statistics file, an L indicates that the variable was available via linkage with other databases

*: Comprises all separate regions for Italy, Belgium, and England/Wales

†: the variable was available, but was not provided to us because of data protection policy

‡: country of birth

In all countries, the cause of death variable was provided as an ICD-10 (3 digits) coded variable, except in The Netherlands and in Italy, where the data protection policies called for certain aggregation. In these countries we negotiated to have 27 pre-determined aggregated cause of death categories, for which we in broad outlines followed the instruction manual by the U.S. Department of Health and Human Services [43].

Besides socio-demographic and clinical variables we also aimed to include a number of residence and healthcare system characteristics. As the municipality (or the parish, council, or local authority) of residence was available on the data files, the variables urbanisation, contextual SES-measures, and number of hospital beds per 1,000 inhabitants were operationalized by linking existing statistics to this place of residence of the deceased.

The Dutch data protection policy, however, did not allow providing us a database containing the municipality of residence of the deceased. At our request the inclusion of the residence characteristics was therefore done in advance by the Dutch Central Bureau of Statistics.

## Discussion

Previous research has demonstrated that death certificates can be a very useful basis to study and monitor the place of death in society [30,32,34-37,39,41,42], and can therefore be a useful public health tool. This study demonstrates that it is feasible to conduct cross-national research on the place of death using death certificate data. However, the suitability of the data files seems to differ between countries, and there are country variations in the difficulty to obtain the necessary data.

While other studies have made cross national comparisons of the method of death certification[44], and while death certificates have been the basis for several cross national comparisons of cause-specific mortality [45], this study is, to our knowledge, the first to make a cross-national evaluation of the suitability of death certificate data regarding place of death. The study only involved seven entire countries and five regions, and can therefore not necessarily be generalized to other countries or regions. Nevertheless the information is ample enough to give some insights into the opportunities and the limitations of using death certificate data to study and monitor the place of death.

### Opportunities

Death certificates have a long tradition as health indicator and as a monitoring tool for public health policy. A major strength is that of completeness: death certificates allow describing patterns within a whole population and not just for a sample. The issue of place of death can be studied across patient populations and across settings, which has been indicated as one of the limitations in many of the previous place of death studies[26]. Our study in nine countries covers more than 1.1 million deaths. This provides more statistical power, potentially leading to more reliable results, and making it is possible to use multivariable statistical models with many associated factors, or to generate meaningful results for specific subpopulations (e.g. lung-cancer or HIV patients, low educated people, specific regions) [42]. As our study demonstrates, most variables on the death certificate data are available for researchers. Especially the Italian and Belgian death certificate data provided many variables besides the place of death. Fewer variables were available directly via the death certificates in other countries, but linkage with other databases made it possible to include several variables, indicated in the literature as relevant to studying place of death [15,19,24,26,33,34,36,46-50]. Linkage could be made with unique identification numbers to include a number of important socio-demographic variables, or via the residence of the deceased to include variables such as health care system statistics or contextual socio-economic status. In principle there is an even larger potential, via linkages with other databases, to include additional relevant information (e.g. hospitalisation and individual healthcare use). This could eventually move us further beyond place of death towards researching care at the end-of-life, and might shape good opportunities for health policy to monitor on how health care resources are allocated within each country and between countries within Europe. However, this possibility and the implications this will have on the procedure to use the data for research, needs further investigation.

Because of the comparability of death certificate data as a study method throughout time and across different countries, they facilitate reliable comparison of results. Comparison of temporal trends of place of death across nations, and placed against other mortality trends (e.g. cause of death, age, living conditions) can for example potentially allow us to monitor the impact of public health policy (e.g. implementation of palliative care services, reorganization of home care).

Finally, an advantage is that the data are relatively easy to obtain and at a relatively low cost price (range of 0 to +/-3500 € in our study).

### Weaknesses

Our study shows that there is considerable variation between countries in the type and comprehensiveness of information on the death certificate data. The place of death variable, while certified in all countries, was in several countries not or only in a limited way coded on the death certificate data files. Therefore several countries only allowed making rough distinctions between dying at home vs. other, or dying in a hospital vs. other. In The Netherlands the variable did not appear on the database before 2003; in Italy only since 2003 in more than 2 categories.

Additionally, procedures to obtain the data and the rules of data-protection were sometimes a barrier to easily get and use the data. The required permissions from different instances often complicated getting the data and integrating them into one data file, resulting in long waiting periods. The privacy and data-protection rules also limited the use of the data.

Finally, next to well known weaknesses of death certificates concerning incorrect cause of death certification, misclassification [51,52] and possible country and time variation in (mis)classifications (which might be a potential impediment for comparisons between countries and over time), another weakness for studying place of death is that death certificate data do not contain all variables regarded as relevant in predicting the place of death [16,46,50,53], such as information about patients' preferred place of death, or qualitative information about the dying process (e.g. characteristics of the course of the disease, the predictability of death, the use or need of a specific therapy). This can perhaps be overcome by linking death certificates to other information (e.g. on the quality of end of life care). In itself, however, death certificate data, primarily having an administrative purpose and only secondary a research purpose, remain not well designed for non-etiological purposes, like monitoring 'good death' or quality of dying [29]. The use of death certificate data reveals statistical patterns, but does not allow us to draw conclusions on the choices, behaviours, attitudes, processes, or feelings that underlie or precede these patterns [30].

## Conclusion

Based on our findings, we believe that death certificate data are certainly a useful tool to give good insight in place of death in relation to other factors in a cross-national perspective and an ideal basis to interpret complementary qualitative and epidemiological studies. However, in order to make international monitoring of place of death more feasible and accurate, improvements are recommended:

1. A minimum set of variables should be (made) available [42]. Next to the place of death variable (with at least the categories hospital, care home, and own home), age, sex, cause of death (in ICD10 codes, or in detailed aggregations), and living situation (e.g. single, in household, in institution) and/or marital status should be minimally available to construct an appropriate statistical model (controlling satisfactory for confounders). This set can be elaborated with other variables (e.g. socio-economic status, hospital bed rate) that are highly relevant to monitor specific target populations and specific associated factors.

2. We suggest therefore to make modifications on aspects of the death certificates, for example by striving for more standardization in the 'place of death' variable, and by regularly modifying the place of death variable according to developments in the patterns of dying in our society (e.g. providing a category of 'hospice'). In the light of monitoring how health care resources are allocated distinguishing patients dying in a regular inpatient facility or in a highly equipped intensive care unit might be opportune. Also modifications in the coding of the death certificates can be considered, for example by the coding of all certified information on place of death.

3. Well-thought-out procedures to link death certificate information with other databases might not only preclude duplicate registration of certain variables, but also possibly increase the reliability of the data (as we can not expect physicians to accurately provide all kinds of information on the patient) and at the same time prevent unnecessarily burdening certain informants (e.g. if personal information about the decedent needs to be provided by a bereaved family member).

4. Finally, we also suggest some modifications in the procedures to get permission to use the death certificate data for research purposes. The collection of death certificates is primarily driven by an administrative purpose. National laws and regulations regarding collection and dissemination of death certificate have often been drafted accordingly and therefore often limit dissemination of these data for other purposes (e.g. research). Increasing privacy measures additionally limit this kind of dissemination of data. Although a thorough scrutinising of an application is surely required in order to protect confidentiality, additional protective requirements should be proportional to the possible harm individuals might suffer from a possible (ab)use of the data [54]. More standardisation in the procedures to get permission to use death certificate data for research purposes is required, and ideally one (centralised) authorisation should be sufficient. This may require including additional specifications for research use of the data in existing laws and regulations. Nevertheless, if the useful opportunity to include information via linkage with other data sources (e.g. discharge records) will be increasingly employed, a good balance will have to be sought between the protection of personal data (and safeguarding of anonymity) on the one hand, and the difficulty of the procedure to get permission for the data on the other hand. To facilitate cross national comparative studies, the manner in which these privacy rules are applied for research can best be discussed in an international context.

## Abbreviations

BE: Belgium

DK: Denmark

IT: Italy

NL: The Netherlands

NO: Norway

SE: Sweden

SES: Socio-economic status

UK: United Kingdom

ICD: International Classification of Diseases

## Competing interests

The author(s) declare that they have no competing interests.

## Authors' contributions

All authors have contributed to the design of the study and to the data-collection in their own country. JC was responsible for the integration of all data and prepared a first draft of this article. All authors have been involved in critically revising the first draft and read and approved the final manuscript.

## Pre-publication history

The pre-publication history for this paper can be accessed here:



## Supplementary Material

Additional File 1 Appendix DCs of all countries. This additional file presents the death certificate forms used to certify deaths (at the time of the study) in Belgium, Denmark, Italy, The Netherlands, Sweden, Norway, England and Wales, and Scotland. Click here for file
