# Supplementary material for: Using death certificate data to study place of death in 9 European countries: opportunities and weaknesses
Source: BMC Public Health. 2007 Oct 8;7:283. doi: 10.1186/1471-2458-7-283 (PMC2099436; doi:10.1186/1471-2458-7-283)
Supplement: Additional File 1 — Appendix DCs of all countries. This additional file presents the death certificate forms used to certify deaths (at the time of the study) in Belgium, Denmark, Italy, The Netherlands, Sweden, Norway, England and Wales, and Scotland. [file 1471-2458-7-283-S1.pdf]

Belgium

Model III C 0102041111074222906

STROOK C

OVERLIJDEN VAN EEN PERSOON VAN EEN JAAR OF OUDER

(Strook in te vullen en onder gesloten omslag te plaatsen door de ***geneesheer****)*

1. Aard van het overlijden

- ☐ natuurlijke oorzaak
- ☐ verkeersongeval
- ☐ ander ongeval
- ☐ zelfmoord
- ☐ doding
- ☐ wordt onderzocht
- ☐ kan niet bepaald worden

2. Indien de doodsoorzaak niet natuurlijk is, beschrijf de omstandigheden

. . . .

. . . .

. . . .

. . . .

Voorbehouden ☐☐☐☐

3. Ongeval

3.1 Plaats van het ongeval

- ☐ openbare weg
- ☐ werkplaats (of school voor kinderen)
- ☐ thuis
- ☐ onbekend
- ☐ andere, preciseer . . . .

. . . .

3.2 Datum en uur van het ongeval

- datum **(DDMMJJJJ)** ☐☐ / ☐☐ / ☐☐☐☐
- uur **(UUMM)** ☐☐ u ☐☐ m

**4. Doodsoorzaak (1)**		**Duur (2)**
**I. Ziekte of aandoening die rechtstreeks de dood tot gevolg had**		
Logische samenhang van de ziekten/aandoeningen die geleid hebben tot de onmiddellijke doodsoorzaak onder a). Bij vermelding van meerdere ziekten de aan het overlijden ten grondslag liggende het laatst opgeven ("oorspronkelijke doodsoorzaak")	a)	
	veroorzaakt door :<br>b)	. . . .
	veroorzaakt door :<br>c)	. . . .
	veroorzaakt door :<br>d)	. . . .
**II. Geassocieerde oorzaken**		
Bij overlijden nog bestaande ziekten en bijzonderheden, welke tot de dood hebben bijgedragen, doch niet met de onder I. genoemde ziekten in causaal verband staan.	e)	. . . .
	f)	. . . .
	g)	. . . .

(1) Hier wordt niet de wijze van overlijden bedoeld zoals bvb. : hartfalen, syncope, enz... maar de ziekte, het trauma of de complicatie die de dood veroorzaakte. Gelieve slechts één oorzaak per lijn te vermelden.
(2) Tijdsinterval (bij benadering) tussen het begin van de ziekte/aandoening en de dood (preciseer zo nodig in minuten, uren, weken of maanden, ...)

Voorbehouden

a) ☐☐☐☐☐ b) ☐☐☐☐☐ c) ☐☐☐☐☐ d) ☐☐☐☐☐

e) ☐☐☐☐☐ f) ☐☐☐☐☐ g) ☐☐☐☐☐

5. Indien de overledene een vrouw is, is ze, in de loop van het jaar vóór haar overlijden :

5.1 Zwanger geweest

☐ ja ☐ neen ☐ onbekend

5.2 Bevallen

☐ ja ☐ neen ☐ onbekend

6. Autopsie/aanvullende onderzoekingen

- ☐ ja, lopend
- ☐ neen
- ☐ ja, voorzien
- ☐ onbekend

7. Was de attesterende geneesheer de behandelende geneesheer ?

☐ ja ☐ neen

Nr. medisch dossier . . . .

Identificatie van de geneesheer

- RIZIV nummer . . . .
- datum (DDMMJJJJ) ☐☐ / ☐☐ / ☐☐☐☐

naam, voornaam
handtekening
stempel

Belgium

Model III C 0102040911074222997

STROOK A

AANGIFTE VAN OVERLIJDEN VAN EEN PERSOON VAN EEN JAAR OF OUDER

*(Strook in te vullen door de **geneesheer** en te bewaren door het gemeentebestuur)*

Naam en voornaam van de overledene ☐☐☐☐☐☐☐☐☐☐☐☐☐☐☐☐☐☐☐☐☐☐

Echtgeno(o)t(e) of weduw(e)(naar) van ☐☐☐☐☐☐☐☐☐☐☐☐☐☐☐☐☐☐☐☐☐☐

Gewone verblijfplaats : gemeente ☐☐☐☐☐☐☐☐☐☐☐☐☐☐☐☐☐☐☐☐☐☐

straat, nr. ☐☐☐☐☐☐☐☐☐☐☐☐☐☐☐☐☐☐☐☐☐☐

Datum **(DDMMJJJJ)** en uur **(UUMM)** van overlijden ☐☐ / ☐☐ / ☐☐☐☐ ☐☐ u ☐☐ m

Adres van overlijden : gemeente ☐☐☐☐☐☐☐☐☐☐☐☐☐☐☐☐☐☐☐☐☐☐

straat, nr ☐☐☐☐☐☐☐☐☐☐☐☐☐☐☐☐☐☐☐☐☐☐

Nummer van de overlijdensakte ☐☐☐☐☐

Geslacht van de overledene

☐ • mannelijk ☐ • vrouwelijk ☐ • onbepaald

Gerechtelijk-geneeskundig bezwaar tegen begrafenis of crematie (1) ☐ ja ☐ neen

Bezwaar tegen schenking van het lichaam (2) ☐ ja ☐ neen

Verplichte onmiddellijke kisting
- in een hermetische kist (3) ☐ ja ☐ neen
- in een gewone kist (4) ☐ ja ☐ neen

Bezwaar tegen eventuele
- crematie (5) ☐ ja ☐ neen
- conserveringstechnieken (6) ☐ ja ☐ neen
- vervoer zonder kist (7) ☐ ja ☐ neen

Risico voor blootstelling aan ioniserende stralen (3) ☐ ja ☐ neen

Ik, die teken, geneesheer (naam, voornaam, nr. in Orde der geneesheren of RIZIV nummer),

....,

verklaar dat ik de ....om..uur heb vastgesteld dat de hierboven genoemde persoon overleden is.

Handtekening en stempel van de geneesheer

(1) Overlijden zeker of vermoedelijk door uitwendige oorzaak (ongeval, zelfmoord, moord of doodslag).
(2) De overledene vormt een risico voor besmetting zoals bepaald sub (3).
(3) A. de overledene leed aan één van de volgende overdraagbare ziekten: cholera, miltvuur, pokken en andere orthopox virusziekten, virale hemorragische koorts.
B. de overledene vormt een risico op radioactieve besmetting (cfr. K.B. van 28 februari 1963 - B.S. 16 mei 1963 - art. 69.4, art. 69. 7 en art. 3).
(4) De overledene leed aan één van de volgende overdraagbare ziekten: AIDS, hondsdolheid, pest, virushepatitis (behalve bevestigde virushepatitis A).
(5) Prothesen die werken op lithiumbatterijen dienen verwijderd, alsook elke andere prothese die radio-elementen bevat.
(6) - cfr (2) en (3)
- slechte toestand van het lijk (ontbonden, verhakkeld)
- overlijden dat zeker of vermoedelijk aan een uitwendige oorzaak te wijten is.
(7) cfr (2) en (3)

Belgium

Model III C

01020410110742229O3

STROOK B

STATISTISCH FORMULIER VOOR HET OVERLIJDEN VAN EEN PERSOON VAN EEN JAAR OF OUDER

*(Strook in te vullen door de **geneesheer**, te verifiëren door het gemeentebestuur en over te maken aan de bevoegde geneesheer-ambtenaar)*

1. Datum en uur van het overlijden

- datum **(DDMMJJJJ)** ☐☐ / ☐☐ / ☐☐☐☐
- uur **(UUMM)** ☐☐ u ☐☐ m

2. Plaats van het overlijden

☐ • thuis ☐ • openbare weg

☐ • ziekenhuis ☐ • werkplaats

☐ • bejaardentehuis

☐ • andere, preciseer . . . .

. . . .

. . . .

3. Geslacht van de overledene

☐ • mannelijk ☐ • vrouwelijk ☐ • onbepaald

Belgium

Model III C 01020412110742229 09

STROOK D

STATISTISCH FORMULIER VOOR HET OVERLIJDEN VAN EEN PERSOON VAN EEN JAAR OF OUDER

*(Strook in te vullen door het **gemeentebestuur** met de hulp van de aangever)*

1. Gemeente (of land) van overlijden

Code N.I.S. ==>

2. Nummer van de overlijdensakte

3. Gemeente (of land) waar de overledene gewoonlijk verbleef

Code N.I.S. ==>

4. Geboortedatum (DDMMJJJJ)

/ /

5. Opleiding (hoogste voltooide opleiding of diploma)

- geen opleiding of lager onvoltooid
- lager onderwijs
- lager middelbaar
  - ⇒ beroeps
  - ⇒ technisch
  - ⇒ algemeen
- hoger middelbaar
  - ⇒ beroeps
  - ⇒ technisch
  - ⇒ algemeen
- hoger van het korte type
- hoger van het lange type of universitair
- onbekend
- andere, preciseer . . . .

. . . .

. . . .

6. Huidige beroepstoestand

- actief(ve)
- student(e)
- werkloos
- gepensioneerd
- invalide
- zonder beroep
- andere, preciseer . . . .

. . . .

. . . .

7. Sociale staat in het laatst uitgeoefend beroep

- zelfstandige
- bediende
- arbeider(ster)
- helper(ster)
- zonder beroep
- onbekend
- andere, preciseer . . . .

. . . .

8. Uitgeoefende beroepen, welke ook de huidige beroepstoestand weze (begin met het laatst uitgeoefend beroep)

- 1.

Code N.I.S. ==>

- 2.

Code N.I.S. ==>

- 3.

Code N.I.S. ==>

9. Nationaliteit van de overledene

Code N.I.S. ==>

10. Burgerlijke staat van de overledene

- ongehuwd
- gehuwd
- weduw(e)(naar)
- uit de echt gescheiden
- van tafel en bed gescheiden
- onbekend

11. Indien de overledene gehuwd was : (DDMMJJJJ)

- geboortedatum van de overlevende echtgeno(o)t(e) / /
- datum van het laatste huwelijk / /

12. Leefde de overledene :

- alleen
- in een privé-huishouden
- in een instelling
- andere, preciseer . . . .

. . . .

. . . .

Denmark

Dødsattest

til lov nr. 402 af 13. juni 1990 om ligsyn, obduktion og transplantation m.v.

Til begravelsesmyndigheden

PERSONNUMMER (CPR nummer)

man Mand — woman Kvinde — stillborn DØDFØDT

For dødfødt angives moderens personnummer

FULDE NAVN: Full name

ERHVERV/Tidligere erhverv: occupation

BOPÆL adresse, kommune, amt: Adress

DØDSSTED adresse, postnr.: Adress for place of death

hospital Sygehus — nursing home/home for the elderly Plejehjem/ beskyttet bolig — own home Eget hjem — other Andet — specification of other Specificer:

FINDESTED hvis dødssted er ukendt: finding place if place of death is unknown

Date of death DØDSDATO — Dag Måned År Time Minut

Finding date if date of death is unknown FINDEDATO, hvis dødsdato er ukendt — Dag Måned År

signs of death DØDSTEGN — Rigor — Livores — Cadaverositas — Maceratio — Andet

A. UNDERTEGNEDE LÆGE

Har den kl. synet liget af ovennævnte og forefundet de ovennævnte dødstegn.

Bekræfter, at der ikke foreligger tilknytningsforhold, som omhandlet i Justitsministeriets bekendtgørelse om lægers adgang til at konstatere dødens indtræden, foretage ligsyn, udstede dødsattest og foretage obduktion § 1.

Har ikke fundet omstændigheder af den i lov om ligsyn, obduktion og transplantation m.v. § 4 stk. 1 nævnte art, og der er efter mit skøn således ikke grund til mistanke om, at døden er forårsaget ved en forbrydelse, jf. Kirkeministeriets bekendtgørelse om begravelse og ligbrænding § 2 stk. 2,2. og 3. punktum.

Here is signed under normal circumstances

Dato — Underskrift

Lægens navn og adresse evt. stempel

B. UNDERTEGNEDE LÆGE

Har den kl. synet liget af ovennævnte og forefundet de ovennævnte dødstegn.

Bekræfter, at der ikke foreligger tilknytningsforhold, som omhandlet i Justitsministeriets bekendtgørelse om lægers adgang til at konstatere dødens indtræden, foretage ligsyn, udstede dødsattest og foretage obduktion § 1.

Har fra politiet modtaget meddelelse om, at stedfunden indberetning efter lov om ligsyn, obduktion og transplantation m.v. § 4 stk, 1. ikke giver anledning til retslægeligt ligsyn, og der er intet til hinder for, at ligbrænding kan finde sted, jf. Kirkeministeriets bekendtgørelse om begravelse og ligbrænding § 2 stk. 2,2. og 3. punktum.

Here is signed if the signing physician has contacted the police e.g. in case of sudden unexpected death and the police has found no reason for further investigation

Dato — Underskrift

Lægens navn og adresse evt. stempel

C. UNDERTEGNEDE EMBEDSLÆGE (RETSLÆGE)

Har ved retslægeligt ligsyn den kl. forefundet dødstegn eller andre forhold der er uforenlige med livets beståen.

Bekræfter, at der ikke foreligger tilknytningsforhold, som omhandlet i Justitsministeriets bekendtgørelse om lægers adgang til at konstatere dødens indtræden, foretage ligsyn, udstede dødsattest og foretage obduktion § 1.

Here is signed by the coroner if a legal investigation has taken place

Dato — Underskrift

Embedslægens (retslægens) navn og adresse evt. stempel

D. POLITIETS PÅTEGNING

Det bekræftes i medfør af lov om ligsyn, obduktion og transplantation m.v. § 7 stk. 2., at der intet er til hinder for at liget begraves, brændes eller - efter udstedelse af ligpas - føres ud af landet.

Here is signed by the police if a legal investigation has taken place

Dato — Underskrift

Politiets stempel

Vejledning: se bagsiden

0163028401

Form. 1431

Til Sundhedsstyrelsen

Denmark

Dødsattest

henhold til lov nr. 402 af 13. juni 1990 om ligsyn, obduktion og transplantation m.v.

PERSONNUMMER (CPR nummer)

Mand (man) Kvinde (woman) DØDFØDT (stillborn)

For dødfødt angives moderens personnummer

FULDE NAVN name

Tidsinterval mellem sygdommens eller skadens opståen og dødens indtræden

DØDSÅRSAG I Det sygdoms- og skadesforløb som førte til døden: cause of death

den umiddelbare dødsårsag var

A Immidiate cause

som var en følge af which was a result of

B

som var en følge af

C

som igen var en følge af (den tilgrundliggende lidelse)

D

DØDSÅRSAG II Andre væsentlige sygdomme, misbrug eller skader, der kan have medvirket til dødens indtræden. Ved misbrug angives misbrugets art.

Factors that may have participated in causing death

DØDSMÅDEN

Naturlig død natural death

Ulykke accident

Selvmord suicide

Drab/Vold killing/violence

Uoplyst unknown

OPERATION FORETAGET

ja

nej

I bekræftende fald, for hvilken lidelse: ~~operation~~

Operationens art: surgery before death

Operationsdato:

OBDUKTION autopsi

retslig Legal

anden other

forbud prohibition

ingen none

Hvis yderligere undersøgelser - specificer hvilke:

Supplerende oplysninger: Other information

Vejledning: se bagsiden

Signature of physician

Dato

Lægens underskrift (skal altid udfyldes)

Det retslægelige ligsyn har fundet sted i forbindelse med (politiets repræsentant stilling, navn og tjenestested)

Lægens navn og adresse evt. stempel

Væsentlige obduktionsfund

Underskrift
(af den læge der har påført obduktionsfundene)

0163028402

Form. 1431

Italy

Cognome e nome del defunto [illegible] Età 72

ISTAT D.4 - Edz. 2001 M.
COPIA PER L'A.S.L.

ISTAT
istituto nazionale di statistica

SCHEDA DI MORTE OLTRE IL 1° ANNO DI VITA PER MASCHIO

PARTE A - A CURA DEL MEDICO

N.B. Per i quesiti seguiti da [illegible] barrare X quello corrispondente al caso.

PROVINCIA DI [illegible] COMUNE DI [illegible]

LUOGO DEL DECESSO
Abitazione ______ 1
Istituto di cura ______ 2
Altro ______ 3

CODICE DELLA A.S.L. DI DECESSO

RISCONTRO DIAGNOSTICO
Richiesto ______ 1
Non richiesto ______ 2

Riservato all'ISTAT: I, II, III, E

MORTE DA CAUSA NATURALE

1. Causa iniziale (Scrivere in stampatello)
Indicare la malattia che, attraverso eventuali complicazioni o stati morbosi intermedi da indicarsi al n.2, ha condotto alla causa terminale, da indicarsi al n.3.

Intervallo tra l'inizio della malattia e la morte (a): anni ___ mesi ___ giorni ___

2. Causa intermedia o complicazione (Scrivere in stampatello)
Indicare l'eventuale complicazione o successione morbosa della malattia indicata al n.1.
anni ___ mesi ___ giorni ___

3. Causa terminale (Scrivere in stampatello)
Indicare la malattia o lo stato morboso che ha direttamente provocato il decesso.
anni ___ mesi ___ giorni ___

4. Altri stati morbosi rilevanti (Scrivere in stampatello)
Indicare le eventuali malattie o gli stati morbosi che hanno contribuito al decesso ma senza rapporto con le cause indicate al n. 1, 2 e 3.
anni ___ mesi ___ giorni ___

[illegible]

Timbro [illegible] indirizzo e telefono del medico o dell'ospedale

MORTE DA CAUSA VIOLENTA

5. Causa violenta
Accidentale 1 — Infortunio sul lavoro 2 X
Suicidio 3 — Omicidio 4

6. Descrizione della lesione (Scrivere in stampatello)

7. Malattie o complicazioni eventualmente sopravvenute a seguito della lesione (Scrivere in stampatello)

8. Stati morbosi preesistenti che hanno eventualmente contribuito al decesso (Scrivere in stampatello)

9.1 Mezzo o modo con quale la lesione è stata determinata (caduta da una scala a piedi, impiccagione, fucilato, ecc.) (Scrivere in stampatello)

9.2 Data dell'accidente, suicidio, omicidio: Giorno / Mese / Anno

9.3 Intervallo tra azione violenta e morte (b): [illegible]

9.4 Luogo dell'accidente, suicidio, omicidio
Casa 1 — Fattoria 2 — Cantiere 3 — Strada o piazza 4
Luogo di ricreazione e sport 5
Altro luogo (specificare) [illegible]

Dichiaro che le cause di morte sopra esposte sono, secondo scienza e coscienza, sono quelle accertate [illegible]
curante / necroscopo

PARTE B - NOTIZIE A CURA DELL'UFFICIALE DI STATO CIVILE (CONTROLLARE ED EVENTUALMENTE CORREGGERE IL COGNOME E IL NOME DEL DEFUNTO)

N.B. - Per i quesiti seguiti da [illegible] barrare X quello corrispondente al caso.

ATTO DI MORTE
Numero 6
Parte 1 [illegible] 2 X
Serie 1 A.B 2 C
UFFICIO DI STATO CIVILE
CIRCOSCRIZIONE ______ (n)

Comune / Provincia: [illegible]

1. Data di morte: [illegible] Giorno / Mese / Anno
2. Data di nascita: 16 / 10 / 1930
3. Località di nascita
Stesso comune di morte 1
Altro comune 2 X — [illegible]
Stato estero 3
4. Età compiuta — Anni 4 [illegible]
5. Stato civile
Celibe 1 X
Coniugato 2
Vedovo 3
Divorziato o già coniugato (a) 4
Separato legalmente 5
Se coniugato o separato legalmente indicare:
l'anno di nascita del coniuge superstite
l'anno di matrimonio
6. Residenza
Stesso comune di morte 1 X
Altro comune 2
Stato estero 3
7. Grado di istruzione
Laurea 1
Diploma universitario o laurea breve 2
Diploma di scuola media superiore 3
Licenza di scuola media inferiore 4
Licenza elementare o nessun titolo 5

8. Professione

9. Condizione professionale o non professionale
Occupato 1 X
Disoccupato 2
In cerca di prima occupazione 3
Ritirato dal lavoro 4
Studente 5
Inabile al lavoro 6
Altro (compreso servizio di leva o servizio civile) 8

10. Posizione nella professione
Per lavoratori autonomi:
Imprenditore o libero professionista 1
Lavoratore in proprio o coadiuvante 2
Altro 3
Per lavoratori dipendenti:
Dirigente o direttivo 4
Impiegato o intermedio 5
Operaio o assimilato 6 X
Altro (apprendista, lav. a domicilio, ecc.) 7

11. Ramo di attività economica
Agricoltura, caccia e pesca 1 X
Industria 2
Commercio, pubblici esercizi, alberghi 3
Pubblica amministrazione e servizi pubblici 4
Altri servizi privati 5

12. Cittadinanza
Italiana
Per nascita 1 X
Acquisita 2
Straniera 3

Codice individuale del deceduto

[illegible]

Netherlands

B Doodsoorzaakverklaring

Centraal Bureau voor de Statistiek

Ministeriële beschikking van 25 april 2002 (Staatscourant 86) ingevolge artikel 12A van de Wet op de lijkbezorging. De Inspecteur-Generaal voor de Gezondheidszorg is belast met het toezicht op de naleving van de invullingsplicht.

*Naam van de overledene **niet vermelden***

a Gemeente van overlijden

Specimen

Plaats van overlijden

- ☐ ziekenhuis
- ☐ psychiatrisch ziekenhuis
- ☐ verpleeghuis
- ☐ verzorgingshuis
- ☐ overige instellingen
- ☐ thuis
- ☐ elders
- ☐ onbekend

b Sectie

- ☐ vindt niet plaats
- ☐ is verricht
- ☐ wordt nog verricht
- ☐ onbekend

Onderzoekingen

Welke onderzoekingen, al dan niet postmortaal, zijn/worden verricht?

(bijv. histologisch of microbiologisch onderzoek)

c Geslacht

- ☐ mannelijk
- ☐ vrouwelijk

Leeftijd

☐ 28 dagen of ouder

geboortedatum (dag, mnd., jaar)

overlijdensdatum (dag, mnd., jaar)

☐ jonger dan 28 dagen

geboortedatum (dag, mnd., jaar) — geboortetijd (uur, min.)

overlijdensdatum (dag, mnd., jaar) — overlijdenstijd (uur, min.)

duur zwangerschap (weken)

geboortegewicht (gram) — lengte (cm)

d Natuurlijke dood *(zie toelichting ommezijde)*

1a Ziekte die ***rechtstreeks*** de dood tot gevolg had — duur tussen begin ziekte en overlijden

1b + c Ziekten die hebben geleid tot de doodsoorzaak onder a. Bij vermelding van meerdere ziekten de aan het overlijden ten grondslag liggende ziekte onder c opgeven.

b veroorzaakt door/gevolg van — duur tussen begin ziekte en overlijden

c veroorzaakt door/gevolg van — duur tussen begin ziekte en overlijden

2 Bijkomstige, bij overlijden nog bestaande ziekten en bijzonderheden die tot de dood hebben bijgedragen, doch niet met de onder 1 genoemde ziekten in causaal verband staan. — duur tussen begin ziekte en overlijden

e Niet-natuurlijke dood, incl. levensbeëindigend handelen

Oorzaak

- ☐ zelfdoding
- ☐ ongeval
- ☐ misdrijf
- ☐ onbekend of anders

Omstandigheden die tot de niet-natuurlijke dood hebben geleid

(bijv. bromfietser door auto aangereden, van trap gevallen, overboord geslagen bij aanvaring, bij stofzuigen door elektrische stroom getroffen, bij grondwerk door zand bedolven, vergiftiging)

Aard van het letsel

(bijv. schedelbasisfractuur, borstkas ingedrukt)

Plaats van de gebeurtenis

(bijv. thuis, in fabriek of op werkplaats, op straat, aan boord, op het land, aan het strand)

Levensbeëindigend handelen

(hiermee wordt niet bedoeld staken of niet-instellen van levensverlengende handelingen, noch pijnbestrijding met als neveneffect eerder overlijden)

- ☐ euthanasie
  (toedienen van middel met uitdrukkelijk doel patiënt te laten overlijden op verzoek patiënt)
- ☐ hulp bij zelfdoding
  (verstrekken of voorschrijven van middel met uitdrukkelijk doel patiënt te laten overlijden op verzoek patiënt)
- ☐ anders

Wat was de onderliggende ziekte? — duur tussen begin ziekte en overlijden

(zie toelichting ommezijde)

deze ruimte niet beschrijven s.v.p.

Bestemd voor de medisch ambtenaar van het Centraal Bureau voor de Statistiek te Voorburg

Netherlands

Specimen

- [ ] mannelijk
- [ ] vrouwelijk

duur zwangerschap
weken

geboortegewicht
gram

datum partus
dag mnd. jaar

Doodsoorzaak

Aangifte is verplicht voor een na een zwangerschapsduur van tenminste 24 weken ter wereld gekomen vrucht (art. 2 en art. 12A van de Wet op de lijkbezorging van 22 mei 1991).

Definitie doodgeborene: de na een zwangerschapsduur van tenminste 24 weken ter wereld gekomen vrucht, welke na de geboorte geen enkel teken van levensverrichting heeft vertoond.

Eventuele complicaties tijdens zwangerschap of bevalling

g Opmerkingen

Deze ruimte is bestemd voor vermelding van eventuele bijzonderheden die nog niet eerder zijn vermeld (lokalisatie en/of histologie van een gezwel, indicatie voor een diagnostische of operatieve ingreep) en verder elke toelichting die van belang kan zijn voor een zo nauwkeurig mogelijke rubricering van de doodsoorzaak, zoals een partus of abortus binnen de 365 dagen voorafgaand aan het overlijden.

h Naam en correspondentie-adres van de geneeskundige die het formulier invult

naam

eventueel naam instelling

specialisme

straat + nummer

postcode plaats

N.B. Ingeval deze doodsoorzaakverklaring niet is afgegeven door de behandelend geneeskundige, hieronder diens personalia vermelden.

naam

eventueel naam instelling

specialisme

straat + nummer

postcode plaats

Als:
- [ ] behandelend geneeskundige
- [ ] gemeentelijk lijkschouwer
- [ ] geneeskundige aangewezen door de Officier van Justitie
- [ ] waarnemer

datum dag mnd. jaar

handtekening

Toelichting *(zie ook www.cbs.nl/doodsoorzaakverklaring)*

vraag d

*In het geval van een natuurlijke dood, dient u onder 1a de ziekte te vermelden die rechtstreeks de dood tot gevolg had. Onder 1b en 1c dienen de ziekten te worden vermeld die hebben geleid tot de onder 1a genoemde doodsoorzaak. Het onderliggend lijden dient het laatst, onder 1c, te worden vermeld. Termen die symptomen aanduiden (bijv. astma cardiale, insufficientia cordis, verlamming, enz.) dienen in ieder geval bij het onderliggend lijden zoveel mogelijk te worden vermeden. Indien u de noodzaak daartoe toch aanwezig acht, gelieve u daarvan een omschrijving te geven onder **g**.*

vraag e

In het geval van levensbeëindigend handelen dient u de onderliggende ziekte in te vullen, zoals in onderstaand voorbeeld 'longcarcinoom'.

	Voorbeeld 1	Voorbeeld 2	Voorbeeld 3
1a	LONGEMBOLIE	LEVERINSUFFICIËNTIE	UREMIE
b	OPERATIE VOOR PANCREATITIS	LEVERMETASTASEN	URINERETENTIE
c		MAAGCARCINOOM	HYPERPLASIE PROSTAAT
2	MYODEGENERATIO CORDIS	ZIEKTE VAN PARKINSON	DIABETES MELLITUS

Voorbeeld 4

- [x] euthanasie

LONGCARCINOOM

deze ruimte niet beschrijven s.v.p.

Fastsatt av Sosialdepartementet 1993

Norway

Legeerklæring om dødsfall/melding om unaturlig dødsfall

Jfr. lov om leger av 13.06.1980 §§ 40 og 41.

Blanketten fylles ut i samsvar med rettledningen på side 2 og leveres rekvirenten (den som har plikt til å melde dødsfallet) i forseglet konvolutt som ikke [illegible] adresseres til skifteretten og på landet til lensmannen på dødsstedet. Kopi av legeerklæringen sendes den lokale politimyndighet, hvis dødsfallet kan være unaturlig. (Se rettledning på side 2.)

Avdødes slektsnavn, for- og mellomnavn	Kjønn	Fødselsnr. (11 siffer)	Fylles ut av Statistisk Sentralbyrå
Bosted, kommune	Gate og husnr.	Postnr. og poststed	
Dødssted, kommune	Død utenfor institusjon: ☐ Hjemme ☐ Annet sted ☐ Under transport til sykehus	☐ Død i sykehus eller annen institusjon	
For døde i sykehus eller annen institusjon: Institusjonens navn		Død (dag, mnd., år)	
Hvis sykehus: Avdeling. For annen institusjon: type institusjon			
Yrke (også eventuelt forsørgerens)			
Ekteskapelig status: ☐ Ugift ☐ Gift ☐ Enke-/mann ☐ Skilt ☐ Separert	For barn døde innen 24 timer etter fødselen, hvor lenge varte livet?	Timer / Minutter	
Navn og adresse på den lege som har behandlet avdøde for den undersøkte sykdom			

Opplysning om dødsårsaken

Alle rubrikker må fylles ut. (Se rettledning på baksiden.)

		Omtrent tid mellom sykdommens begynnelse og døden
I. Sykdom eller tilstand som direkte (umiddelbart) har ført til døden. (Her skal ikke føres dødsmåten f.eks. hjertesvikt, hjertelammelse, asteni, men den sykdom, skade eller komplikasjon som umiddelbart fremkalte døden.)<br><br>Oppgi den eller de sykelige tilstander, skader eller misdannelser som har ført til (lå bak) den dødsårsak som er anført ovenfor. Den tilstand som innledet sykdomsforløpet føres sist.	a)<br>Som skyldtes (var en følge av)<br>b)<br>Som skyldtes (var en følge av)<br>c)	
II. Andre vesentlige tilstander som kan ha bidratt til dødens inntreden, men som ikke står i direkte årsaksforhold til den sykdom eller tilstand som har fremkalt døden.		

Dersom døden skyldtes skade (ulykke) eller følger av denne:	Dato skaden (ulykken) skjedde	Sted: ☐ (Ved) Hjemmet ☐ Annet sted	Yrkesulykke: ☐ Ja ☐ Nei
	Hvordan skjedde ulykken		

Spesielle omstendigheter ved dødsfallet/forsett [illegible] (sett kryss)

☐ Drap ☐ Selvmord ☐ Misbruk av medisiner ☐ Medisinsk feil ☐ Ukjent årsak ☐ Plutselig uventet ☐ Dødsfall i fengsel/arrest ☐ Ukjent lik ☐ Yrkessykdom

Ble det foretatt operasjon? ☐ Ja ☐ Nei	Dato operert	Tilstand [illegible]
Opplysninger under punkt I bygger på: ☐ Obduksjon ☐ Undersøkelse før døden ☐ Synsing av liket	Vil den oppgitte dødsårsak senere bli revurdert? ☐ Ja ☐ Nei ☐ Vet ikke	

Undertegnede lege som har synet liket og som har behandlet den døde under siste sykdom (sett et kryss), erklærer herved at dødsårsaken er den ovenfor nevnte.

☐

Dato ____ Lege ____

Adresse:

Melding om unaturlig dødsfall er sendt [illegible] til politimyndigheten

☐ Ja ☐ Nei

Undertegnede lege erklærer herved at det ikke er grunn til å anta at døden er voldt ved en straffbar handling. (Erklæringen gir [illegible] likets [illegible])

Dato ____ Lege ____

Adresse:

I. Forsvirt [illegible] lensmannen og sendes den offentlige lege/politiet på dødsstedet	II. Forsvirt politiet og sendes den offentlige lege på dødsstedet	III. Off. legeskaftsråd (stempel)
Dato ____ For skifteretten/lensmannen ____<br>Adresse:	Dato ____ For politimesteren ____<br>Adresse:	

Sweden

Var dödsorsaksintyget utfärdas (klinik, mottagning etc. samt adress, telefonnr och ev. telefaxnr) ❶

Rensa

DÖDSORSAKSINTYG

Personuppgifter (efter- och förnamn, adress) ❶❷

Den avlidnes personnummer (12 siffror) ❸

För barn under 7 dygn anges ålder

☐ Dödfött ☐ Under 24 tim ☐ 24 tim t.o.m. 6 dygn

Dödsdatum

☐ Säkert ☐ Sannolikt

Datum (8 siffror) år, mån, dag — Klockslag (t.ex. 06:20)

Typ av dödsplats ❹

☐ Akutsjukhus ☐ Sjukhem eller annan vårdbostad ☐ Privat bostad ☐ Annan/okänd

LÄKARENS UTLÅTANDE OM DÖDSORSAKEN

☐ **Intyget ersätter ett tidigare utfärdat**

I Det sjukdoms- eller skadeförlopp som lett till döden ①

A	**Den terminala dödsorsaken var**	**Ungefärlig debut eller varaktighet**
B	**som var en följd av**	**Ungefärlig debut eller varaktighet**
C	**som i sin tur var en följd av**	**Ungefärlig debut eller varaktighet**
D	**som i sin tur var en följd av**	**Ungefärlig debut eller varaktighet**

II Andra sjukdomar och skador som bidragit till dödsfallet (ange även ungefärlig debut eller varaktighet) ②

Dödsorsaksuppgifterna grundas på

☐ Klinisk obduktion ③ ☐ Undersökning före döden ☐ Yttre likbesiktning ☐ Rättsmedicinsk obduktion ☐ Rättsmedicinsk likbesiktning

Opererad inom 4 veckor före döden ④

☐ Ja ☐ Nej ☐ Uppgift saknas

Operationsdatum (8 siffror)

Tillstånd som föranledde ingreppet

Vid missbruksrelaterat dödsfall, ange typ av drog/droger ⑤

☐ Alkohol ☐ Läkemedel ☐ Narkotika ☐ Annat, ange vad:

Vid skada/förgiftning, ange orsak och eventuell avsikt ⑥

☐ Olycksfall ☐ Avsiktligt självtillfogad ☐ Avsiktligt tillfogad av annan ☐ Oklart om avsikt förelegat

Datum för skada/förgiftning

Kort beskrivning av hur skadan/förgiftningen uppkommit ⑦

Plats för skadehändelsen ⑧

☐ Bostad/ bostadsområde ☐ Skola, offentlig lokal, institutionsområde ☐ Idrotts-/motions-anläggning ☐ Gata/väg ☐ Lantbruk/ industriområde ☐ Annan/okänd

Aktivitet vid skadetillfället

☐ Idrott/motion ☐ Fritidsverksamhet/lek ☐ Förvärvsarbete ☐ Utbildning ☐ Annan/okänd

UNDERSKRIFT AV UTFÄRDANDE LÄKARE

Ort	Datum (8 siffror) år, mån, dag	Läkarens namnteckning	
Tjänsteställe	Telefonnr (inkl. riktnr)	Namnförtydligande	Befattning

SoSB 76016 2003-06 PEE

Socialstyrelsen

Sweden

Hur man fyller i blanketten

❶ För klinik- och personuppgifter kan tydliga avtryck med stämpelbrickor godtas.

❷ För dödfött barn eller barn som avlidit inom 28 dygn efter födseln skall inte stämpelbricka användas. Ange kön och eventuellt namn. Ange dessutom moderns personnummer, namn och adress.

❸ Den avlidnes personnummer måste fyllas i, även för barn som avlidit inom 28 dygn efter födseln. Ange födelsedatum, om personnummer inte finns.

❹ Typ av dödsplats
- Akutsjukhus: sjukhus för somatisk eller psykiatrisk korttidsvård
- Sjukhem eller annan vårdbostad: innefattar äldrebostäder och servicehus med tillgång till vård- och omsorgspersonal, gruppboenden, gruppbostäder, institutioner etc.
- Privat bostad: utesluter sådant eget boende som ingår i föregående kategori.

① Uppgifter om dödsorsak används huvudsakligen i medicinsk forskning. Ange därför diagnosen så specifikt som möjligt. Undvik förkortningar. Skriv inte kodnummer. Uppgifter om debut eller varaktighet är mycket väsentliga för en korrekt klassificering av dödsorsaken.

Ange för
- tumör: malign, benign samt primärtumörens läge,
- diabetes mellitus: typ och komplikationer,
- njursjukdomar: komplikationer (t.ex. hypertoni), etiologi till njursvikt.

Ange det sjukdoms- eller skadeförlopp som haft störst betydelse för dödsfallet. Skriv *en* diagnos per rad och ange debut eller varaktighet.

Om förloppet kan beskrivas med en enda diagnos anges denna på rad A. Rad B–D lämnas då tomma. Krävs flera diagnoser för att beskriva förloppet skrivs den terminala dödsorsaken på rad A. Det eller de tillstånd som ledde fram till den terminala dödsorsaken skrivs på raderna nedanför. Det tillstånd som inledde förloppet skrivs nederst, så att det bildas en kedja av orsaker och komplikationer från det inledande tillståndet på den nedersta raden upp till den terminala dödsorsaken på rad A. Akuta patologiska förlopp kan ingå i en sådan kedja men också att ett tidigare tillstånd berett vägen för ett senare, t.ex. genom vävnadsskador eller funktionsnedsättning.

För hjärtsvikt och lunginflammation anges eventuella bakomliggande tillstånd, t.ex.
- rad A: hjärtsvikt; rad B: lårbensbrott,
- rad A: lunginflammation; rad B: influensa.

Socialstyrelsen begär ofta komplettering av dödsorsaksintyg som verkar ofullständigt ifyllda. Ange därför tydligt om någon uppgift inte kan lämnas, t.ex.
- rad A: hjärtsvikt, rad B: okänt (patienten avböjde utredning).

② Sjukdomarna anges i rangordning efter den betydelse de haft för dödsfallet, med den allvarligaste sjukdomen först. Ange tlllståndens debut eller varaktighet inom parentes efter varje diagnos.

③ Om obduktion görs, fylls inte uppgifterna om dödsorsaken i förrän obduktionsresultatet föreligger.

④ Som operation räknas åtgärder enligt Socialstyrelsens ”Klassifikation av kirurgiska åtgärder 1997”.

⑤ Ett dödsfall anses vara missbruksrelaterat om drogmissbruk uppenbarligen framkallat eller bidragit till dödsfallet, t.ex. genom akut förgiftning, beteenderubbningar eller långtidsskador.

Vid missbruk av narkotiska läkemedel upptagna i FASS väljs alternativet ”läkemedel”. Vid missbruk av preparat som normalt inte har medicinsk användning, t.ex. amfetamin, väljs alternativet ”narkotika”. Flera rutor kan fyllas i, t.ex. vid blandmissbruk.

⑥ Välj det alternativ som är mest sannolikt även om omständigheterna inte är klarlagda. Alternativet ”Oklart om avsikt förelegat” väljs endast om man inte kan avgöra om olycksfall eller avsiktligt tillfogad skada är det mest sannolika uppkomstsättet.

⑦ Ange för
- trafikolycka: om den döde var t.ex. förare, passagerare eller fotgängare och vilka typer av fordon som var inblandade (personbil, lastbil, motorcykel etc.),
- förgiftning: vilken eller vilka substanser,
- fall: t.ex. i trappa, från stege, halkat på mattkant,
- drunkning: naturlig eller konstgjord vattensamling (t.ex. vattendrag, sjö, brunn, badkar), fall ned i vatten (t.ex. genom brunnslock) eller på annat sätt (fått kramp under simtur).

⑧ För definition av plats och aktivitet vid skadetillfället hänvisas till avsnitten om plats- och aktivitetskoder i ”Kapitel XX” i Socialstyrelsens ”Klassifikationer av sjukdomar och hälsoproblem 1997”.

Information

I begravningslagen (1990:1144), begravningsförordningen (1990:1147) och i Socialstyrelsens föreskrifter och allmänna råd (SOSFS 1996:29) Vissa åtgärder inom hälso- och sjukvården vid dödsfall finns bestämmelser om vilka åtgärder som skall vidtas vid dödsfall.

Den läkare som har utfärdat dödsbeviset skall även ansvara för att dödsorsaksintyget utfärdas. När den läkare som har utfärdat dödsbeviset begär det, är den läkare som har vårdat den avlidne för den sjukdom eller det tillstånd som ledde till dödsfallet skyldig att utfärda dödsorsaksintyget (16 § begravningsförordningen). Den läkare som utfärdar dödsorsaksintyget får inte vara make, barn, förälder, syskon eller på något annat sätt närstående till den avlidne (4 kap. 2 § begravningslagen).

Dödsorsaksintyget skall sändas in till **Dödsorsaksstatistiken, Socialstyrelsen, 106 30 Stockholm,** ***inom tre veckor*** från det att dödsbeviset utfärdades. Om tidsgränsen tre veckor undantagsvis behöver överskridas, skall Socialstyrelsen skriftligen underrättas om förseningen och skälen för denna (16 § begravningsförordningen).

Den läkare som har utfärdat dödsorsaksintyget skall behålla en tydligt läsbar kopia av handlingen. Kopian skall behandlas som en journalhandling (SOSFS 1996:29, avsnitt 4.3, jämförd med 5 § patientjournallagen (1985:562)).

Om en rättsmedicinsk undersökning har gjorts, svarar Rättsmedicinalverket för att dödsorsaksintyget utfärdas och sänds in (16 § begravningsförordningen).

Om polismyndigheten beslutar att någon rättsmedicinsk undersökning inte skall göras, skall myndigheten underrätta den läkare som har lämnat dödsbeviset om detta (4 kap. 4 § begravningslagen). Den läkare som har lämnat dödsbeviset ansvarar då för att dödsorsaksintyget utfärdas och kan från polisen få sådana uppgifter som behövs för intyget, t.ex. uppgifter om omständigheterna vid dödsfallet (SOSFS 1996:29, avsnitt 9.3).

England/Wales

RIAR 002952

CERTIFIED COPY OF AN ENTRY
Pursuant to the Births and Deaths Registration Act 1953

DEATH		Entry No.
Registration district	Administrative area	
Sub-district		
1. Date and place of death		
2. Name and surname	3. Sex	
	4. Maiden surname of woman who has married	
5. Date and place of birth		
6. Occupation and usual address		
7.(a) Name and surname of informant	(b) Qualification	
(c) Usual address		
8. I certify that the particulars given by me above are true to the best of my knowledge and belief	Signature of informant	
9. Cause of death		
10. Date of registration	11. Signature of registrar	

SPECIMEN

Certified to be a true copy of an entry in a register in my custody.

SPECIMEN

*Superintendent Registrar
*Registrar

Date

**Strike out whichever does not apply*

CAUTION: THERE ARE OFFENCES RELATING TO FALSIFYING OR ALTERING A CERTIFICATE AND USING OR POSSESSING A FALSE CERTIFICATE. © CROWN COPYRIGHT

WARNING: A CERTIFICATE IS NOT EVIDENCE OF IDENTITY.

Scotland

SCHEDULE 2 **Regulation 3**

Medical certificate of cause of death

Form 11

(Section 24(1) of the Registration of Births, Deaths, and Marriages (Scotland) Act 1965)

F(11)0000001

The completed certificate is to be taken to the Registrar of Births, Deaths and Marriages

For registration office use
Year: ....
RD number: ....
Entry number: ....

Name of deceased ....

Date of death

Day		Month		Year			

Time of death
Fill in an approximate time if you do not know the exact time *(Please use the 24-hour clock)*

Hour		Minute	

Place of death ....

Cause of death

I hereby certify that to the best of my knowledge and belief, the cause of death was as stated below:

Approximate interval between onset and death (Years Months Days)

I Disease or condition directly leading to death*

(a) .... | Years | Months | Days

due to (or as a consequence of)

Antecedent causes
Morbid conditions, if any, giving rise to the above cause, stating the underlying condition last

(b) .... | Years | Months | Days

due to (or as a consequence of)

(c) .... | Years | Months | Days

due to (or as a consequence of)

(d) .... | Years | Months | Days

II Other significant conditions contributing to the death, but not related to the disease or condition causing it

.... | Years | Months | Days

.... | Years | Months | Days

* This does not mean mode of dying, such as heart or respiratory failure; it means the disease, injury or complication that caused death.

Please tick the relevant box

Post mortem

PM1 ☐ Post mortem has been done and information is included above

or

PM2 ☐ Post mortem information may be available later

or

PM3 ☐ No post mortem is being done

Procurator fiscal

PF ☐ This death has been reported to the procurator fiscal

Extra information for statistical purposes

X ☐ I may later be able to supply the Registrar General with additional information

Attendance on deceased

A1 ☐ I was in attendance upon the deceased during last illness

A2 ☐ I was not in attendance upon the deceased during last illness: the doctor who was is unable to provide the certificate

A3 ☐ No doctor was in attendance on the deceased

Maternal deaths

M1 ☐ Death during pregnancy or within 42 days of the pregnancy ending

M2 ☐ Death between 43 days and 12 months after the end of pregnancy

Signature: ....

Date: ....

Name in BLOCK CAPITALS: ....

....

Official address:

Registered medical qualifications

For a death in hospital
Name of the consultant responsible for deceased as a patient

The doctor has given you this form so that you can arrange for the death to be registered. Once the death is registered, the local registrar will keep this form, but can advise you what other documents you may need and can issue extracts of the entry in the register of deaths

Who should tell the local registrar about the death

One of the following people must go to the registration office and tell the local registrar about the death.

- Any relative of the deceased, or
- any person present when the person died, or
- the deceased's executor or other legal representative, or
- the occupier of the property where the person died,

or if there is no such person,

- anyone else who knows the information to be registered.

Where to take the form

In Scotland, a death may be registered

- either in the registration district where the person died
- or in the registration district where the deceased lived (the district of "usual residence") if that was in Scotland.

Usual residence means the deceased's permanent home, not at an address such as a holiday address where he or she may have been staying at the time of death.

If you need advice about what to do with the form, please telephone any local registrar in Scotland (see 'Registration of Births, Death and Marriages' in the telephone book).
